# The characteristics of newly diagnosed adult early-onset diabetes: a population-based cross-sectional study

**DOI:** 10.1038/srep46534

**Published:** 2017-04-19

**Authors:** Xiantong Zou, Xianghai Zhou, Linong Ji, Wenying Yang, Juming Lu, Jianping Weng, Weiping Jia, Zhongyan Shan, Jie Liu, Haoming Tian, Qiuhe Ji, Dalong Zhu, Jiapu Ge, Lixiang Lin, Li Chen, Xiaohui Guo, Zhigang Zhao, Qiang Li, Zhiguang Zhou

**Affiliations:** 1 Peking University People’s Hospital, Beijing, China; 2 China-Japan Friendship Hospital, Beijing, China; 3 Chinese People’s Liberation Army General Hospital, Beijing, China; 4 Sun Yat-sen University Third Hospital, Guangzhou, China; 5 Shanghai Jiaotong University Affiliated Sixth People’s Hospital, Shanghai, China; 6 First Affiliated Hospital, Chinese Medical University, Liaoning, China; 7 Shanxi Province People’s Hospital, Taiyuan, Shanxi, China; 8 West China Hospital, Sichuan University, Chengdu, Sichuan, China; 9 Xijing Hospital, Fourth Military Medical University, Xi’an, Shanxi, China; 10 Affiliated Drum Tower Hospital of Nanjing University Medical School, Nanjing, Jiangsu, China; 11 Xinjiang Uygur Autonomous Region’s Hospital, Urumqi, Xinjiang, China; 12 Fujian Provincial Hospital, Fuzhou, Fujiang, China; 13 Qilu Hospital of Shandong University, Jinan, Shandong, China; 14 Peking University First Hospital, Beijing, China; 15 Henan Province People’s Hospital, Zhengzhou, Henan, China; 16 Second Affiliated Hospital of Harbin Medical University, Harbin, Heilongjiang, China; 17 Xiangya Second Hospital, Changsha, Hunan, China.

## Abstract

To investigate the characteristics of newly diagnosed early-onset diabetes in the Chinese population, 2801 newly diagnosed diabetes participants without known diabetes or pre-diabetes in a national cross-sectional survey were analysed. Participants were divided into quartiles (22–43, 44–52, 53–61 and >61 years) according to age of diabetes onset and the first group were defined as early-onset diabetes group. Early-onset diabetes group had lower systolic blood pressure (SBP), total cholesterol, low density lipoprotein cholesterol, 2-hour post prandial blood glucose and urine albumin creatinine ratio. There was no difference in body mass index, Homeostasis model assessment (HOMA) of beta cell function and diabetes family history between early-onset diabetes participants and any other age groups. HOMA of insulin resistance (IR) scores and disposition index 30 minutes after glucose load (DI_30_) were increased in early-onset diabetes participants. The beta cell function declination was more deteriorated in early-onset diabetes participants. Male gender, triglycerides, HOMA-IR and DI_30_ were positively associated with an earlier age at diagnosis. In conclusion, patients diagnosed with diabetes at a younger age are characterized by a similar cardiovascular risk profile with increased insulin resistance and more severe beta cell failure than patients diagnosed at a later age.

Early-onset diabetes was defined as diabetes with an age of diagnosis at a young age, and different cut-offs for age at diagnosis were used to define early-onset diabetes (30–45 years of age). Although type 2 diabetes is a disease related to aging, the prevalence of adult early-onset type has increased globally^1^,^2^,^3^. A higher proportion of early-onset diabetes was observed more frequently in Asian countries than in Western countries^4^,^5^. The number of early-onset diabetes cases increased 4-fold from 1997 to 2010 in China, and the number of cases will increase by at least 20% over the next 20 years^2^,^5^. Therefore, early-onset diabetes is becoming one of the major health burdens in China.

Recent studies demonstrated that adult early-onset type 2 diabetes is a more progressive disease from a cardiovascular standpoint than late-onset type 2 diabetes^6^,^7^,^8^. These patients lose up to 15 years of life expectancy, which is two-fold greater than the number of years lost by patients with late-onset diabetes^9^. In contrast to late-onset diabetes, early-onset diabetes is associated with a higher risk of developing cardiovascular and microvascular complications, which are largely due to prolonged disease exposure in both Westerners and Asians^3^,^8^,^10^,^11^. A large number of early-onset diabetes patients receive inadequate anti-glycaemic treatment and suboptimal cardio-protective treatment, which may be partially attributed to the lack of knowledge regarding the harm of early-onset diabetes in daily clinical practice^3^,^12^.

The detection of the characteristics of and risk factors for early-onset diabetes is fundamental to the development of better strategies for the prevention and management of this disease. Studies in hospital settings indicate that early-onset diabetes patients have more extensive family history of diabetes, and higher body mass index (BMI), Haemoglobin A1c (HbA1c), and low-density lipoprotein cholesterol (LDL-C) level in Western countries and Asia^13^,^14^, although there are disputes regarding the significance of these differences^15^. Large hospital based studies in China suggested early-onset diabetes participants had lower SBP, lower LDL-C, higher HbA1c and similar BMI and triglyceride levels compared with late-onset diabetes participants^3^. Selection bias in hospital-based studies is usually inevitable, particularly when using healthy participants who are recruited from hospitals as controls. Applying the study results from a hospital-based study to the general population, and also to diabetes at an elementary stage is also difficult^16^. Understanding the pathophysiology of newly diagnosed early-onset diabetes is useful for clinical decision making. It was reported that insulin resistance assessed by HOMA-IR was independently positively associated with age in newly diagnosed diabetes^17^. Beta cells function declines by 50–60% prior to T2DM diagnosis and the reduction happens before hyperglycaemia is present according to The UK Prospective Diabetes Study (UKPDS)^18^. However, whether the beta cell function in adult newly diagnosed early-onset diabetes participants is more impaired than late-onset diabetes is unknown. There are no population-based studies that address the clinical and pathophysiological characteristics of newly diagnosed early-onset diabetes patients. The current study aimed to explore the characteristics of and potential risk factors for newly diagnosed early-onset diabetes using the data from the China National Diabetes and Metabolic Disorders Study.

## Methods

The China National Diabetes and Metabolic Disorders Study was a national cross-sectional survey of Chinese participants aged 20–75 years conducted from June 2007 to May 2008. This study recruited 47,325 subjects (18,976 men and 28,349 women) using a complex multistage stratified sampling method according to geographical region, economic development status, and degree of urbanization. Complete oral glucose tolerance test (OGTT) data and demographic information were collected in 46,239 adults. 43,864 participants without previously diagnosed diabetes or pre-diabetes, as defined by self-reporting anti-diabetes treatment or a diabetes or pre-diabetes history, were included in the final analysis. The design, protocol, and major epidemiological findings of this study have been previously published^19^.

The institutional review board or independent ethics committee of each participating institution approved this study protocol. The institutes included Peking University People’s Hospital, Beijing, China-Japan Friendship Hospital, Beijing, Chinese People’s Liberation Army General Hospital, Beijing, Sun Yat-sen University Third Hospital, Guangzhou, Shanghai Jiaotong University Affiliated Sixth People’s Hospital, Shanghai, First Affiliated Hospital, Chinese Medical University, Liaoning, Shanxi Province People’s Hospital, Taiyuan, Shanxi, West China Hospital, Sichuan University, Chengdu, Sichuan, Xijing Hospital, Fourth Military Medical University, Xi’an, Shaanxi, Affiliated Drum Tower Hospital of Nanjing University Medical School, Nanjing, Jiangsu, Xinjiang Uygur Autonomous Region’s Hospital, Urumqi, Xinjiang, Fujian Provincial Hospital, Fuzhou, Fujiang, Qilu Hospital of Shandong University, Jinan, Shandong, Peking University First Hospital, Beijing, Henan Province People’s Hospital, Zhengzhou, Henan, Second Affiliated Hospital of Harbin Medical University, Harbin, Heilongjiang and Xiangya Second Hospital, Changsha, Hunan. All methods were performed in accordance with the relevant guidelines, and informed consent was obtained from each subject prior to data collection.

Trained and qualified physicians completed questionnaire surveys and physical examinations in person during on-site surveys. Each subject completed a standard questionnaire on demographic characteristics, lifestyle, and medical history. Annual family income was categorized as less than 10,000 RMB or 10,000 RMB and greater. Education was divided into two groups according to the highest degree achieved by participants, namely, college education or higher and high school and lower. Smoking status was divided into current smokers who smoked every day and non-current smokers who never smoked or smoked previously or occasionally. Information on family history of diabetes, cardiovascular disease, and hypertension was collected based on the medical history of first-degree relatives (e.g., parents, siblings, and children). Height and weight were measured using a height-weight scale with subjects standing in bare feet in light clothing. Body mass index (BMI) was calculated as weight divided by height squared. Waist circumference was measured as the minimum circumference between the subcostal margin and the iliac crest. Blood pressure was measured twice with a 30-second interval between measurements using a mercury sphygmomanometer, and the mean pressure was recorded.

Participants were instructed to avoid excessive exercise and diet control for at least 3 days prior to the OGTT. Participants with no self-reported history of diabetes or diabetes treatments received a 75-g glucose solution (200 ml) after an overnight fast. Blood specimens were collected for insulin and glucose tests after fasting and 30 and 120 minutes after a glucose load or meal.

Plasma glucose was measured using the hexokinase method. Fasting total cholesterol (CHO), triglyceride (TG), high density lipoprotein cholesterol (HDL-C), and low density lipoprotein cholesterol (LDL-C) were measured enzymatically in each biochemical laboratory that met national or provincial quality standards. Specimens were transported on ice to the Central Laboratory of Endocrinology at the China-Japan Friendship Hospital for serum insulin level measurements using radioactive immunoassays. Random urine creatinine and microprotein were measured in the Central Laboratory using the picric acid method and enzymatic method, respectively. The urine albumin to creatinine ratio (ACR) was calculated as the urine microprotein (mg/L) divided by urinary creatinine (g/L).

### Variable definition

Diabetes was defined as a FPG level ≥7.0 mmol/L or a 2 hPG ≥11.1 mmol/L according to the World Health Organization (WHO) diagnostic criteria of 1999^20^. The following two parameters were calculated to evaluate insulin resistance: (1) the HOMA-IR, which primarily indicates hepatic insulin resistance, was calculated as FIN (mU/ml) × FPG (mmol/L)/22.5^7^; and (2) the Matsuda insulin sensitivity index (Matsuda index), which represents whole-body insulin sensitivity, was calculated as 10,000/ ((18 × FPG (mmol/L) × FIN (mU/L) × OGTT average glucose (mmol/L) × OGTT mean insulin (μU/ml))^1/2^ 21. Beta cell function and insulin release were assessed as follows: (1) the HOMA-B: 20 FIN (μU/ml)/(FPG (mmol/L)-3.5); (2) the insulinogenic index (ΔI30/ΔG30), which reflects early-phase insulin secretion after glucose challenge: (INS30 - FIN) (μU/ml)/(PG30 - FPG) (mmol/L), where INS30 and PG 30 refer to insulin and glucose levels 30 minutes after the glucose load, respectively; (3) glucose-corrected insulin area under the curve, which was the glucose-adjusted insulin release: InsAuc30/GluAuc 30 = [FIN (mU/L) + INS30 (mU/L)]/[FPG (mmol/L) + PG30 (mmol/L)] and InsAuc120/GluAuc120 = [FIN (mU/L) + 4 × INS30 (mU/L) + 3 × INS120 (mU/L)]/[FPG(mmol/L) + 4 × PG30 (mmol/L) + 3 × 2 hPG (mmol/L)], where 2 hPG and INS120 refer to glucose and insulin levels 2 hours after glucose loading, respectively; and 4) the disposition index (DI), which indicates the insulin secretion corrected by insulin sensitivity: DI_30_ = InsAuc30 × Matsuda index and DI_120_ = InsAuc120 × Matsuda index^22^. The decline in beta cell function and DI in early- and late-onset diabetes participants was compared with that in their non-diabetes peers in the same age groups. For example, the percentage of HOMA-B decline in early-onset diabetes participants (A) and young non-diabetes participants (B) was evaluated using the equation (B-A)/B*100.

### Statistical analysis

Statistical analysis was performed using SPSS for Windows 19.0 software (SPSS Inc., Chicago, IL, USA). Newly diagnosed diabetes participants were divided into quartiles: diabetes onset at 20–43 years, 44–52 years, 53–61 years and more than 61 years. The first age group was defined as early-onset diabetes and the last group was defined as late-onset diabetes. Continuous variables are presented as the mean (95% confidence interval [CI]) or geometric means (95% CI). Categorical data are presented as numbers (%). Differences in mean values between groups were tested using analysis of variance (UNI-ANOVA) with Bonferroni correction. The chi-square test followed by post hot Bonferroni test was used to assess the differences between frequencies. The criteria for significance of multiple comparison was adjusted using Bonferroni correction. Binary logistic regression using 3 different models was used to assess the odds ratio for each risk factor for early-onset diabetes in reference to late-onset diabetes. The criteria for significance for regression was adjusted using Bonferroni correction.

## Results

A total of 2,801 participants were diagnosed with diabetes based on the results of the oral glucose tolerance test (OGTT) in 43,864 adults aged 20 years and older. Among all the newly diagnosed diabetes participants, 654 (23.3%) participants were classified as having early-onset diabetes (age < = 43 years).

### Characteristics of newly diagnosed early-onset diabetes participants

Newly diagnosed early-onset diabetes participants included a higher proportion of males and high income participants than participants who had diabetes onset at 53 years or later. They had more well educated participants than any other age group. The prevalence of cardiovascular disease (CVD) was lower in the early-onset diabetes group compared with participants who had diabetes onset at 53 years or later. There was no difference in family history of hypertension, diabetes and CVD between early-onset diabetes participants and any of the other three age groups. The BMI was similar across all 4 age groups after adjusting for sex, but early-onset diabetes participants had smaller waist circumstances than participants who had diabetes onset at 44 years or later. Early onset diabetes participants had the lowest SBP and DBP among all age groups and lower total cholesterol and LDL-C levels compared with participants had diabetes onset at 53 years or later. There was no difference in TG levels between early diabetes participants and any other age groups. The urine ACR were decreased in early-onset diabetes referring to late-onset diabetes participants. Early-onset diabetes participants had a lower 2-hour postprandial plasma glucose (2 hPG) and higher FPG. Early onset diabetes participants had higher fasting insulin than participants with diabetes onset between 44–61 years and lower 2-hour postprandial insulin (IN2h) values than late-onset diabetes participants. A higher percentage of diabetes diagnoses were made based on a FPG value ≥7.1 mmol/L alone and a lower percentage of diagnoses were made based on a 2 hPG value ≥11.1 mmol/L alone in early-onset diabetes participants. Higher HOMA-IR and DI_30_, and unaltered DI_120_ levels, Matsuda index and HOMA-B values were observed in the early-onset diabetes participants (Table 1).

### The change in insulin secretion and beta cell function in early- onset diabetes participants

The declination of insulin secretion and beta cell function was enhanced in early-onset diabetes compared with participants had diabetes onset later than 44 years. DI_30_ decreased by 67.4% (65.5%, 65.3%), 65.3% (63.5%, 67.1%), 65.3% (63.5%, 67.1%) and 63.5% (61.6%, 65.4%) in the early-onset diabetes participants, participants with diabetes onset between 44–52 years, participants with diabetes onset between 53–61 years and late-onset diabetes participants, respectively (p = 0.04). DI_120_ decreased by 65.7% (63.8%, 67.6%), 61.7% (59.8%, 63.6%), 62.8 (60.9, 64.6%) and 60.9 (59.0%, 62.8%) in the four consecutive age groups (p = 0.003). Beta cell function as assessed by HOMA-B decreased by 59.6% (50.5%, 68.8%), 40.9% (31.9%, 49.8%), 40.5% (31.6%, 49.5%) and 44.3% (35.3%, 53.3%) (p = 0.01).

### Risk factors for participants with early-onset diabetes compared with participants with diabetes onset after 44 years

A logistic regression analysis using different models was performed to calculate the odds ratio for developing early-onset diabetes compared with participants with diabetes onset at 44 years or later. After adjusting for cardiovascular risk factors and metabolic parameters, male gender resulted in an increased odds ratio (OR) for diabetes diagnosis at an earlier age. Per standard deviation (SD) elevation of triglyceride level, HOMA-IR and DI_30_ values increased the odds ratio for early-onset diabetes by 15%, 17% and 24%, respectively. Per SD increase of SBP led to an approximate 50% decrease in the OR for early-onset diabetes. BMI, current smoking, diabetes family history were not independent early-onset diabetes risk factors. (Table 2).

## Discussion

This large, multi-centre, cross-sectional, population-based study in China revealed that the occurrence of early-onset diabetes was 23.3% among newly diagnosed diabetes patients. Early-onset diabetes participants were characterized by lower blood pressure and LDL-C levels, similar BMI, triglycerides and HOMA-B values, increased HOMA-IR and DI_30_ values and a more profound loss of beta cell function than late-onset diabetes and/or middle-age-onset participants. Male gender, higher triglycerides, higher HOMA-IR, higher DI_30_, and lower SBP were independently associated with a diabetes diagnosis at an earlier age.

This study suggested there was a high prevalence of newly-diagnosed diabetes participants in China. This is consistent with another large population based study, the China Non-communicable Disease Surveillance 2010 survey^5^, which revealed that newly diabetes participants aged 18–40 years accounted for 15.7% of all newly diagnosed diabetes cases as determined by the OGTT. This proportion is much higher than the IDF-predicted values in Australia, the UK, and Japan^2^. These alarming findings from the two large-scale population-based epidemiological studies in China indicated that diabetes in the young population had reached pandemic proportions. An imminent and effective action for the prevention of diabetes in young people should be implemented in this country.

Our results that early-onset diabetes participants had a similar ‘cardio-risk’ phenotype to middle-age and late-onset diabetes participants. This was partly inconsistent with other studies, which were all hospital based studies, demonstrating that early-onset diabetes had a similar or adverse cardiovascular profile. Although the genetics associated with type 2 diabetes was different between Westerners and Asians^2^, this discrepancy may not be completely due to ethnicity difference among these studies. Unchanged BMI, decreased LDL-C, increased blood glucose levels and reduced SBP was found not only in our study, but also in American newly diagnosed early-onset diabetes participants from Kaiser Permanente Northwest Division (KPNW) and Chinese early-onset diabetes participants from the China National HbA1C Surveillance System^3^,^7^. However, Joint Asia Diabetes Evaluation (JADE) study, which recruited 41,029 Asian participants and 10% of them were from China, suggested the BMI, LDL-C and TG levels were higher in early-onset diabetes participants^13^.

Sample selection could be one explanation for the discrepancy between our study and other studies which was the difference between hospital- and population-based studies. Hospital-based recruitment may be limited to people who can afford healthcare and those with severe symptoms. In contrast, population-based screening is advantageous for identifying individuals with less severe cases of diabetes and covering areas with a shortage of medical resources. By this means, our study has the increased external validity related to reduced recruitment bias.

Another reason for the discrepancy could be the length of risk factor exposure in these participants. Early-onset diabetes participants had a 100% increase in macrovascular complications^23^ and a 69% increase in microvascular complications^11^ at any given age, and this risk was greatly associated with disease duration^11^,^24^. It is possible that even the cardiovascular risk factors were similar between early-onset and late-onset diabetes, the exposure of these risk factors were much longer in early-onset diabetes due to longer life expectancy in these people. In fact, the atherosclerosis progression was determined by the same factors including smoking, hypertension, obesity and dyslipidaemia in both young and old individuals^25^. As a cross-sectional study, we were unable to measure the exposure period of these risk factors and how they contribute the the disease progression.

Young age is a remarkable protective factor for cardiovascular disease and cardiovascular risk factors such as high blood pressure, dyslipidaemia, obesity and high blood glucose^26^. Our study suggested these protective effects of youth were at least partly lost in early-onset diabetes participants. The absence of the ‘cardio-protective’ phenotype in newly diagnosed early-onset diabetes patients puts these patients at the same risk of developing cardiovascular disease as the middle-age-onset or late-onset diabetes participants. More social and clinical attention is necessary to develop policies and prevention and management strategies for participants with early-onset diabetes who have had the disease for a prolonged period of time.

Although age per se is an independent factor negatively associated with beta cell function in non-diabetes participants^27^, beta cell function, as indicated by HOMA-B, was unaltered among different age groups in this study. The early-onset diabetes had increased first phase insulin release but similar late phase insulin release with late-onset diabetes. Similarly, clamp studies with small sample size suggested there was no difference in either early phase nor late phase insulin release between middle-aged and old diabetes participants^28^. Our study also demonstrated that the magnitude of the decline in beta cell function was greater in early-onset diabetes than in middle age onset and late-onset diabetes when age-matched non-diabetes peers were used as a reference. Our study also has the advantage of being able to compare the characteristics of early-onset diabetes participants with their non-diabetes peers in the appropriate context because of complete data collection. This is consistent with previous findings that beta cell function declined more rapidly in early-onset diabetes (15% per year)^29^ later onset subjects (6% per year)^30^. A more severe decline in beta cell function may cause a quicker switch from oral anti-diabetes drugs to insulin injections in early-onset diabetes participants. This explains why early-onset type 2 diabetes was associated with a higher proportion of participants receiving insulin injections, even after adjustment was made for disease duration^13^.

A higher proportion of early-onset diabetes participants elevated fasting glucose levels alone than diabetes participants with disease onset at 44 years or later. This result supported the finding that hepatic insulin resistance, as indicated by HOMA-IR, was elevated in early-onset diabetes patients in this study because fasting hyperglycaemia is highly associated with hepatic insulin resistance^31^. However, the Matsuda index, which reflects both hepatic and muscle insulin sensitivity^32^, was unaltered among all age groups possibly because the muscle insulin sensitivity was similar between the two patient groups. This result was in contrast to previous findings that a mild positive association between age and HOMA-IR was detected in newly diagnosed diabetes participants in UKPDS^17^. Again, this could be owing to the different recruitment strategy between our study and UKPDS. Another possible reason for this result may involve an increase in inflammation, fatty liver, which are other determinants of insulin resistance in addition to age, BMI, and lipid levels^33^, in early-onset diabetes Chinese individuals.

Previous studies suggested that a family history of diabetes greatly increased the odds ratio for developing diabetes at an earlier age^34^. Our study suggested that diabetes family history was not significantly different between early- and other participants. A study using the same data set demonstrated that the risk of development of diabetes increased as the number of first-degree relatives with diabetes increased for any given participant. However, this risk increased evenly in both young and old participants^35^.

There are some limitations of this study. First, because of the cross-sectional design of this study, the clinical implication of the observed characteristics in early-onset diabetes participants is not known. Prospective cohort studies are necessary to observe the natural history of newly diagnosed early-onset diabetes patients. Second, the impact of aetiological heterogeneity on the clinical characteristics of early-onset diabetes in our study could not be addressed because of the lack of investigation using factors such as autoimmune markers. However, the prevalence of latent autoimmune diabetes in adults (LADA) was relatively low (5.9%) and consistent across different age groups^36^. Additionally, the impact of classic type 1 diabetes on our study could be ignored because classic type 1 diabetes is a relatively rare condition in the Chinese population^37^, and this condition is more likely to present in a clinical practice setting with classic symptoms, such as polydipsia, polyuria, weight loss, and ketonuria or ketone acidosis. Third, patients with known diabetes were excluded from this study because of incomplete data, leading to a shortage of participants with severe disease conditions and a long disease duration. Also, as a cross-sectional study, we were unable to include the length of exposure of all the cardiovascular risk factors. Further cohort studies involving these patients should be initiated to fill this knowledge gap.

## Conclusion

Although newly diagnosed early-onset diabetes participants are more progressive to cardiovascular disease, our study suggested newly diagnosed early-onset diabetes had a similar cardiovascular risk profile with newly diagnosed middle-age and late- onset diabetes. Younger age at diabetes diagnosis was associated with increased insulin resistance and accelerated beta cell failure. The difference in the cardiovascular profiles of early-onset diabetes participants between our study and other hospital based studies may be owning to different sample recruitment strategies. Our study shed new light on the clinical characteristics of early-onset diabetes as a population based study.

## Additional Information

**How to cite this article:** Zou, X. *et al*. The characteristics of newly diagnosed adult early-onset diabetes: a population-based cross-sectional study. *Sci. Rep.* **7**, 46534; doi: 10.1038/srep46534 (2017).

**Publisher's note:** Springer Nature remains neutral with regard to jurisdictional claims in published maps and institutional affiliations.

## Figures and Tables

**Table 1:** Characteristics of quartile groups of newly diagnosed diabetes participants.

Age category	20–43	44–52	53–61	>61	P	Significance
N	654	713	723	711		
Age (year)	36 (35, 36)	48 (47, 48)***	57 (57, 57)***	68 (68, 69)***	<3*E-5	†††
Male (%)	338 (51.7%)	346 (48.5%)	290 (40.1%)*	308 (43.3%)*	<3*E-5	†††
College education^¶^	186 (28.5%)	130 (18.3%)*	67 (9.4%)*	73 (10.4%)*	<3*E-5	†††
Income >10,000 RMB/year^¶^	382 (65.2%)	408 (60.4%)	369 (54.7%)*	362 (55.1%)*	<3*E-5	†††
Current CVD^¶^	8 (1.3%)	11 (1.6%)	32 (4.7%)*	79 (11.7%)*	<3*E-5	†††
Smoking history^¶^	170 (26.1%)	193 (27.1%)	126 (17.5%)*	128 (18.1%)*	<3*E-5	†††
HBP family history^¶^	250 (44.1%)	314 (50.2%)	280 (45.8%)	211 (37.9%)	0.0004	†
DM family history^¶^	194 (33.7%)	233 (35.8%)	184 (28.4%)	189 (30.3%)	0.02	NS
CVD family history^¶^	125 (22.3%)	164 (26.3%)	148 (24.8%)	107 (19.5%)	<3*E-5	†††
**Cardio**-**metabolic risk factors**						
WC (cm)^¶^	86 (85, 87)	88 (87, 89)***	89 (88, 90)***	89 (88, 89)***	<3*E-5	†††
BMI (kg/m2)^¶^	25.8 (25.5, 26.1)	26.2 (25.9, 26.5)	26.1 (25.8, 26.4)	25.6 (25.3, 25.8)	0.008	NS
SBP (mmHg)	124 (122, 125)	130 (129, 131)***	137 (136, 139)***	142 (141, 144)***	<3*E-5	†††
DBP (mmHg)	81 (80, 82)	84 (83, 85)***	85 (84, 86)***	82 (81, 83)	<3*E-5	†††
Total cholesterol (mmol/L)^¶^	5.0 (4.9, 5.1)	5.1 (5.0, 5.2)	5.3 (5.2, 5.4)***	5.3 (5.2, 5.4)***	<3*E-5	†††
HDL-C (mmol/L)^¶^	1.29 (1.26, 1.31)	1.32 (1.29, 1.34)	1.31 (1.28, 1.33)	1.34 (1.32, 1.37)*	0.031	NS
LDL-C (mmol/L)^¶^	2.85 (2.77, 2.93)	2.99 (2.92, 3.07)	3.10 (3.02, 3.18)***	3.13 (3.06, 3.21)***	<3*E-5	†††
TG (mmol/L)^#,¶^	1.8 (1.7, 1.8)	1.8 (1.7, 1.8)	1.9 (1.8, 1.9)	1.7 (1.6, 1.7)	0.006	NS
ACR (mg/g)^#,¶^	17 (15, 17)	17 (15, 17)	19 (17, 21)	23 (21, 25)***	<3*E-5	†††
**Insulin resistance and beta cell function markers**						
FPG (mmol/L)	7.9 (7.7, 8.1)	7.9 (7.7, 8.1)	7.7 (7.5, 7.9)	7.5 (7.5, 7.7)*	0.005	NS
2 hPG (mmol/L)	12.9 (12.5, 13.3)	13.8 (13.5, 14.2)**	14.1 (13.8, 14.5)***	14.2 (13.8, 14.2)***	<3*E-5	†††
Diagnosed using FPG only	205 (31.3%)	142 (19.9%)*	122 (16.9%)*	106 (14.9%)*	<3*E-5	†††
Diagnosed using 2 hPG only	225 (34.4%	278 (39.0%)	326 (45.1%)*	348 (48.9%)*		
FPG and 2 hPG both met the diagnostic criteria	224 (34.3%)	293 (41.1%)	275 (38.0%)	257 (36.1%)		
FIN (mU/l)^#^	9.9 (9.4, 10.4)	8.8 (8.4, 9.3)**	8.9 (8.6, 9.4)*	9.1 (8.7, 9.5)	0.006	NS
IN2h (mU/l)^#^	34.6 (32.2, 37.2)	37.1 (34.6, 39.9)	36.4 (33.9, 39.1)	40.9 (38.1, 44.0)*	0.008	NS
HOMA-IR^#^	3.3 (3.1, 3.5)	3.0 (2.8, 3.1)*	2.9 (2.8, 3.1)**	2.9 (2.8, 3.1)*	0.003	NS
HOMA-B	55.1 (43.1, 67.2)	68.1 (57.3, 80.8)	66.8 (55.0, 78.5)	68.0 (56.2, 79.9)	0.388	NS
Matsuda	19.4 (18.4, 20.4)	19.7 (18.8, 20.7)	20.2 (19.2, 21.2)	19.5 (18.5, 20.5)	0.71	NS
DI_30_^#,¶^	34.3 (32.6, 36.1)	30.7 (29.1, 32.3)*	30.5 (29.0, 32.1)*	30.5 (28.9, 32.1)*	0.003	NS
DI_120_^#,¶^	45.1 (42.6, 47.9)	42.4 (39.9, 44.9)	41.0 (38.7, 43.6)	42.5 (40.0, 45.1)	0.158	NS

Continuous data are expressed as the mean (95% confidential intervals), calculated using one-way analysis of variance (UNI-ANOVA) after adjusting for sex. The significance was tested using UNI-ANOVA adjusted for sex and the difference between early-onset group and other age groups was detected using Bonferroni post-hoc test. Categorical data were expressed as numbers (%). The significance was tested using chi-square test and following Bonferroni post hot test. For continuous data, *p < 0.05, **p < 0.01, ***p < 0.001 versus early-onset group. p < 0.0016 and †††p < 3*E-5 for one-way ANOVA or Chi-square test (the criteria for significance was adjusted using Bonferroni correction). ^#^Geometric mean; WC: waist circumference; CVD: cardiovascular disease; HBP: high blood pressure; DI: disposition index of 30 minutes or 120 mintues; HOMA-IR: homeostasis model assessment of insulin resistance; HOMA-B: homeostasis model assessment of beta cell function; CHO: cholesterol; LDL-C: low-density lipoprotein cholesterol; HDL-C: high-density lipoprotein cholesterol; FPG and PH2h: fasting and 2-hour prandial plasma glucose, respectively; FIN and IN2h: fasting insulin and 2-hour prandial insulin, respectively. ACR: urine albumin to creatinine ratio. College education: with college or higher degree. Income: annual family income more than 10,000 RMB. ^¶^With missing value.

**Table 2:** The odds ratio of metabolic parameters for early-onset diabetes participants versus non-early-onset diabetes participants.

	Model 1	p	Model 2	p	Model 3	p
Male	1.36 (1.14, 1.62)^†††^	0.001	1.39 (1.12, 1.73)^†^	0.003	1.43 (1,11, 1.85)^†^	0.001
Current smoker			0.98 (0.76, 1.26)	0.995	0.91 (0.67, 1.23)	0.522
SBP			0.45 (0.40, 0.50)^†††^	<1*E-4	0.48 (0.42, 0.55)^†††^	<1*E-4
HDL			0.97 (0.88, 1.07)	0.538	0.98 (0.87, 1.10)	0.732
Triglycerides			1.14 (1.06, 1.22)^††^	0.0003	1.15 (1.06, 1.25)^†^	0.001
BMI			1.12 (1.02, 1.23)	0.024	1.13 (1.01, 1.27)	0.041
With DM family history					1.17 (0.92, 1.48)	0.202
HOMA-IR					1.17 (1.05, 1.30)^†^	0.003
DI_30_					1.23 (1.10, 1.37)^†††^	<1*E-4

The odds ratio (OR) and 95% confidence interval of each parameter versus late-onset diabetes were analysed using binary logistic regression. Continuous variables were divided by the standard deviation before inclusion in the equation. Model 1 included gender. ^†^p < 0.05. Model 2 included Model 1 plus cardiovascular risk factors such as smoking status, body mass index (BMI) systolic blood pressure (SBP), high-density lipoprotein cholesterol (HDL), and triglycerides (n = 2697). ^†^p < 0.0083 and ^††^p < 0.0016 ^†††^p < 1.7*E-4 (criteria for significance was adjusted using Bonferroni correction). Model 3 incorporated Model 2 plus metabolic parameters including diabetes (DM) family history, homeostasis model assessment of insulin resistance (HOMA-IR), and deposition index at 30 minutes after glucose loading (DI_30_) (n = 1843). ^†^p < 0.0056, ^††^p < 0.0001 and ^†††^p < 1*E-4 (criteria for significance was adjusted using Bonferroni correction).
